# A Review of Data Engineering in United States Healthcare Infrastructure

**DOI:** 10.3390/healthcare14101401

**Published:** 2026-05-20

**Authors:** Elizabeth A. Trader, Sahar Hooshmand, Paniz Abedin, Jaeyoung Park, Varadraj Gurupur

**Affiliations:** 1 Department of Electrical and Computer Engineering, University of Central Florida, 4000 Central Florid Blvd, Orlando, FL 32816, USA; elizabeth.trader@ucf.edu; 2 Department of Computer Science, California State University Dominguez Hills, 1000 E. Victoria Street, Carson, CA 90747, USA; 3 Department of Computer Science, Florida Polytechnic University, 4700 Research Way, Lakeland, FL 33805, USA; pabedin@floridapoly.edu; 4 Center for Decision Support Systems and Informatics, University of Central Florida, 4000 Central Florid Blvd, Orlando, FL 32816, USA; jaeyoung.park@ucf.edu; 5 School of Global Health Management and Informatics, University of Central Florida, 4000 Central Florid Blvd, Orlando, FL 32816, USA; varadraj.gurupur@ucf.edu

**Keywords:** healthcare data engineering, electronic health records (EHRs), artificial intelligence, machine learning, data integration, health informatics

## Abstract

With the rapid advancements in artificial intelligence (AI) and machine learning (ML), the role of data engineering has become increasingly critical due to the growing demands for high-quality, large-scale, and well-structured datasets required to train reliable predictive models. Healthcare is one of the most data-intensive industries and has demonstrated strong potential for AI-driven automation in clinical decision support, diagnostics, and operational efficiency. However, healthcare data is often fragmented across multiple systems, inconsistently formatted, and constrained by privacy and regulatory requirements, creating significant barriers to scalable AI adoption. In this review, we examine recent research on healthcare data engineering and AI applications, focusing on how data pipelines, interoperability, and governance frameworks support or limit real-world deployment. This review examined 68 peer-reviewed studies published between 2018 and 2026 across multiple clinical domains, including oncology, cardiovascular disease, infectious disease, neurological disorders, medical imaging, and algorithmic frameworks for explainability and fairness. The reviewed literature shows that while AI models achieve promising performance across these domains, the lack of standardized data architectures and interoperable infrastructure remains a primary bottleneck. The purpose of this study is to highlight key challenges and emerging solutions in healthcare data engineering and outline the future directions needed to support safe, scalable, and trustworthy AI integration in the United States healthcare system. The intended core contributions of this article are to: (i) identify the need for reliable AI systems for healthcare, (ii) explore challenges associated with implementing AI systems in healthcare from a data engineer’s perspective, and (iii) analyze key limitations of data engineering as it applies to the implementation of AI systems in healthcare. It must be noted that one of the key limitations of this narrative review is that the authors mostly used citations from MDPI journals.

## 1. Introduction

The rapid advancements in artificial intelligence (AI) and machine learning (ML) are reshaping industries, and at the heart of this transformation lies the crucial role of data engineering. As AI and ML models become increasingly sophisticated, the demands on data quality, volume, and structure grow exponentially. These models require meticulously curated and well-structured datasets to effectively learn, adapt, and perform an expanding array of complex tasks that researchers and industries are eager to automate. Among the sectors expressing a heightened need for improved data management and automation, the healthcare industry stands out due to its inherent complexity, vast amounts of data, and the critical importance of accuracy and efficiency.

Healthcare systems generate massive volumes of data daily, ranging from patient records and clinical trial data to imaging and genomic information. The challenge lies not only in managing this data but in transforming it into actionable insights that can drive decision-making and improve patient outcomes. Effective data engineering is essential to handle these vast and varied datasets and enable AI and ML models to perform complex tasks that the industry is hoping to automate. This includes but is not limited to accurately predicting diagnoses, recommending treatments, and streamlining administrative processes, among other applications. This review explores the real-world applications of data engineering within the healthcare sector, focusing on how current research and technological advancements can address the pressing demand for automation. The authors delve into various strategies and frameworks that can be leveraged to optimize data pipelines, ensure data integrity, and, ultimately, enhance the deployment of AI-driven solutions in healthcare settings, demonstrating the critical intersection between data engineering and healthcare innovation.

While the healthcare system is uniquely poised to leverage vast amounts of data to effectively implement AI and ML solutions, it also contains unique roadblocks due to the nature of the industry. Unlike some industries that have rapidly adopted AI, the healthcare industry has the risk of serious injury or death if something goes wrong. This introduces heavy liability associated with these applications as well as ethical and legal conundrums surrounding who is responsible for this liability when models fail to perform. Additionally, even though institutions in the healthcare industry may have vast amounts of data, this does not necessarily mean that the data is organized or accessible enough to be used in training. When considering the use of this data for this purpose, these institutions need to consider factors such as privacy laws and potential lawsuits that could arise if something is found during the analysis of the data that indicates bias or discrimination. Based on these observations, the authors felt the need to review this problem based on the following objectives:

- Assessing the Role of Data Engineering in AI and ML Implementation in the Healthcare Industry: Evaluating how data engineering supports the deployment of AI and ML models in the healthcare sector, focusing on the unique data requirements for high-performance, reliable, and scalable AI solutions.
- Identifing Challenges in Healthcare Data Management: Investigating the specific challenges healthcare institutions face in managing, structuring, and utilizing large volumes of data, including issues related to data quality, accessibility, integration, and regulatory compliance.
- Assessing the Future Potential and Limitations of AI in Healthcare: Critically evaluating the future trajectory of AI and ML in healthcare, including potential breakthroughs and ongoing limitations, with a focus on how advancements in data engineering can address existing roadblocks and accelerate adoption.

By accomplishing these tasks, the authors ultimately hope to answer the following question: Is data engineering being employed in the healthcare industry in a way that effectively meets the needs of the industry and its patients? Our observation indicates that the existing review articles focused on AI/ML applied medical science fail to discuss important aspects of data engineering as it applies to reliability, privacy, and interoperability. It is our goal to present this much-needed discussion.

## 2. Background

The use of data engineering in the healthcare industry requires many considerations and has many stakeholders, including but not limited to patients, hospitals and medical centers, insurance companies, pharmaceutical companies, and regulatory agencies such as the Food and Drug Administration and licensure boards. While all of these stakeholders may be generally working towards the same goal of patient wellness, each one of these entities has other considerations that they need to balance with that goal that affects how they weigh patient well-being against other consequences associated with any given decision.

### 2.1. Data Engineering

Data engineering is the process of developing and maintaining systems to collect, organize, and analyze data with the intent of making the data accessible and useful for other applications. These applications can include decision-making made by humans, such as businesses attempting to determine the best path forward, or decision-making made by computers, such as when developing predictive models. Data engineering differs from simply creating ontologies or datasets in that data engineering involves the use and application of data that necessitates maintenance and evolution of the ontology or dataset. While data engineering may include the development of ontologies, ontologies are independent of specific applications [1]. Data engineering is becoming increasingly relevant as more industries, like the healthcare industry, acquire Big Data. Big Data can be defined as information that is too large, unstructured, or fast to be analyzed in a meaningful manner using traditional data processing methods [2]. For example, in the healthcare industry, things such as doctor’s notes in patient health records are not necessarily easily processed. Consider, for example, a doctor who hand-writes a note. While there is most likely an abundance of these notes in any given medical center, unless someone types it into the computer after the fact, the format is not easily machine-readable. Additionally, even if a hospital were to type the notes immediately, how do you efficiently translate human language that may contain colloquialisms, connotations, and malapropisms into something that a computer can utilize?

### 2.2. Data Gaps in Healthcare

Medical misdiagnosis occurs when a healthcare provider provides an inaccurate assessment of a patient’s condition. An inaccurate diagnosis may result in negative consequences for the patient, such as unnecessary medical expenses or death [3]. The consequences of misdiagnosis extend beyond the patient. Healthcare providers and insurance companies are also affected by medical misdiagnosis. With limited data relating to this topic, it can be difficult to quantify the extent of the repercussions incurred by medical misdiagnosis, from financial costs to the patient-and-doctor relationship.

### 2.3. Machine Learning in Healthcare

**Figure 1:**
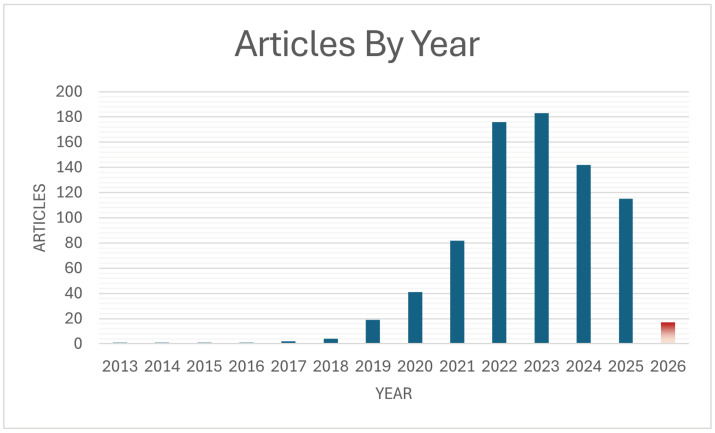
Global articles for machine learning for medical diagnosis indexed in MDPI journals. This bar chart shows the rise in the use of machine learning to identify ailments and aid in the diagnostic process, based on a search of MDPI publications, with 2026 being shown in a different color due to this review being written during that year.

There has been an increasing interest in the use of machine learning to help aid the diagnostic process by researchers. A search of research articles from MDPI that were found using the search prompt "machine learning for medical diagnosis" shows that there was a sharp increase in the number of articles published between the years of 2000 and 2026 at the time of the finalization of this paper. This can be seen in Figure 1, where the final year is colored in red, as this paper was written during the year 2026 and therefore does not have complete data for that year. There were also no published research articles before 2013.

## 3. Materials and Methods

### 3.1. Overview of the Literature Review

**Figure 2:**
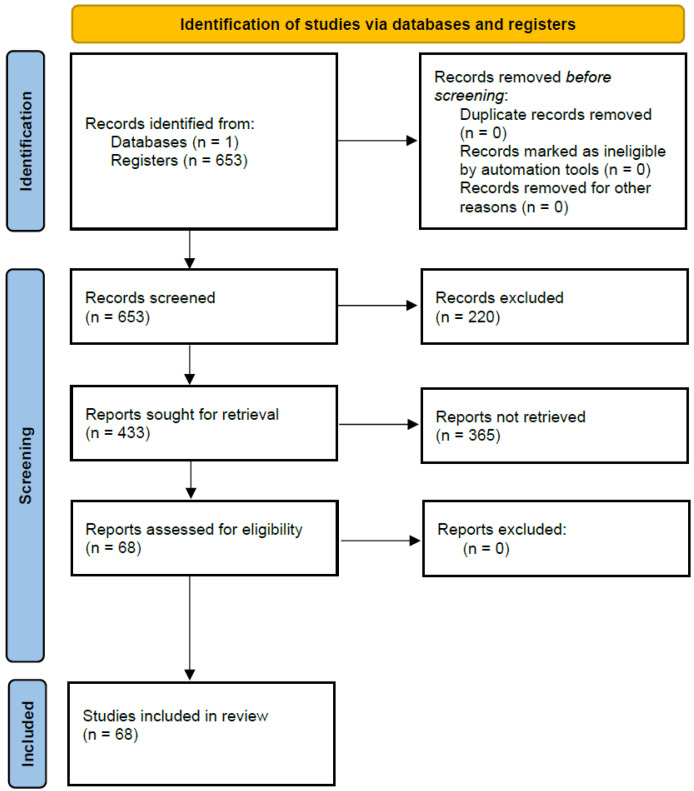
PRISMA flow diagram illustrating the study selection process.

To establish a comprehensive understanding of how artificial intelligence (AI) has been applied to medical diagnostics and clinical data analysis, we performed a structured review of MDPI publications between 2018 and 2026. Searches were executed across multiple MDPI journals, including *Cancers*, *Diagnostics*, *Bioengineering*, *Applied Sciences*, *Sensors*, and *Healthcare*, using the terms “*machine learning*”, “*artificial intelligence*”, “*deep learning*”, “*EHR*”, “*diagnosis*”, and “*predictive analytics*”. Each selected study was grouped according to its primary medical domain, algorithmic framework, and data modality. Thematic synthesis was used to derive categories of AI application: oncology, cardiovascular disease, infectious disease, neurological and mental health disorders, medical imaging innovations, and methodological frameworks for explainability and bias mitigation. To ensure transparency and reproducibility, explicit inclusion and exclusion criteria were applied during the study selection process. Studies were included if they were published between 2018 and 2026 in MDPI journals and applied AI or ML techniques to a healthcare or clinical domain. This review was intentionally scoped to MDPI journals to ensure a consistent standard of peer review and open-access availability across all included studies. This focus also allowed for a manageable and comparable corpus of literature, given the breadth of clinical domains covered, while still capturing a representative range of AI applications in healthcare. The full study selection process is illustrated in Figure 2.

### 3.2. AI in Oncology Research

Oncology remains the most represented field of AI adoption across MDPI journals. These studies demonstrate how deep learning, radiomics, and hybrid feature extraction methods improve cancer detection and classification accuracy.

The study by Ruini et al. [4] developed a convolutional neural network (CNN) pipeline for identifying squamous cell carcinoma using ex vivo confocal laser scanning microscopy. Keywords such as *digital pathology* and *reflectance confocal microscopy* highlight how imaging data was transformed into labeled datasets for automated histologic interpretation. This approach demonstrated the potential of digital staining as a substitute for traditional biopsy slides, thereby accelerating intraoperative cancer diagnosis. More broadly, Jang and Lee [5] reviewed AI-driven digital pathology systems and showed how multimodal deep learning can improve cancer diagnostics and precision oncology workflows.

Additional advances in skin cancer detection have been reported by Naeem et al. [6], which integrated deep learning-based features using dermoscopy images in the SNC_Net architecture. Furthermore, Mobiny et al. [7] proposed a risk-aware machine learning classifier employing Bayesian deep networks and Monte Carlo dropout to address model uncertainty in skin lesion diagnosis, emphasizing physician-friendly interpretability.

A study by Liu et al. [8] analyzed statistical design principles for clinical trials involving AI-assisted breast cancer diagnostic devices. It introduced generalized estimating equations to determine sample size adequacy for algorithm validation, addressing reproducibility and concordance metrics between human and machine assessments. Similarly, Kuno et al. [9] reviewed how AI is increasingly embedded in clinical oncology practice, improving workflow efficiency while enabling new directions in treatment discovery.

The application of radiomics and machine learning to multiparametric breast MRI was explored by Daimiel Naranjo et al. [10], demonstrating improved diagnostic accuracy through dynamic contrast-enhanced MRI and diffusion-weighted imaging. A comparison of computer-aided diagnosis schemes using radiomics versus deep transfer learning methods was presented by Danala et al. [11], highlighting the trade-offs in feature extraction strategies for breast lesion classification. Adebiyi et al. [12] contributed a linear discriminant analysis and classification model combining random forest and support vector approaches for breast cancer diagnosis. Challenges in algorithmic fairness were addressed by Soltan and Washington [13], which examined post-processing methods such as equalized odds to reduce bias in breast cancer stage classification using deep learning. A recent review in *Biomedicines* [14] summarized major AI trends in oncology, emphasizing deep learning, radiomics-based diagnosis, and the growing importance of multimodal clinical integration.

Two further examples emphasize multi-omics integration. Troisi et al. [15] proposed a metabolomics-based screening method for colorectal cancer, combining fecal occult blood tests with ensemble machine learning classifiers. Meanwhile, Mostafa et al. [16] used demographic and biochemical variables to predict hepatic disease progression, demonstrating the importance of structured EHR and laboratory data in building statistical learning models. In addition, Heinzelmann and Piraino [17] discussed how AI-enhanced patient-derived cancer organoids can support precision oncology by improving treatment response prediction and personalized therapeutic evaluation.

An additional study by Sultan et al. [18] investigated whether sequential ultrasound images from the same object could be used for training machine learning models in liver disease detection, addressing concerns about data independence in radiomics.

Prediction of gastrointestinal tract cancers using longitudinal electronic health record data was explored by Read et al. [19], emphasizing temporal feature extraction for early detection. Automated classification of lung cancer subtypes using deep learning and CT-scan-based radiomic analysis was presented by Dunn et al. [20]. Automated lung nodule detection and classification combining deep learning with multiple strategies, including clinical biomarkers and wireless body area networks, was reported by Nasrullah et al. [21]. Supporting these findings, Wang et al. [22] provided meta-analytical evidence that deep learning-based lung nodule detection systems achieve strong early screening performance in thoracic oncology applications. Similar AI-driven computer-aided diagnosis approaches have also been extended beyond thoracic imaging to other organs and cancer types. For example, a comprehensive computer-assisted diagnosis system for early assessment of renal cancer tumors using contrast-enhanced CT was developed by Shehata et al. [23], integrating morphology, texture, and functionality features.

Glioma tumor classification using deep neural network-based features with SVM classifiers was investigated by Latif et al. [24]. Robust, AI-driven segmentation of glioblastoma T1c and FLAIR MRI series was presented by Barhoumi et al. [25], demonstrating low variability using the MRIMath© platform with high Sørensen-Dice scores. A comparative analysis of a novel conditional deep convolutional neural network model using DCGAN-generated synthetic and augmented brain tumor datasets for image classification was reported by Onakpojeruo et al. [26].

Wang et al. [27] improved multi-tumor biomarker health check-up tests using machine learning algorithms for cancer screening. Homogeneous ensemble feature selection methods for mass spectrometry data prediction in ovarian cancer studies were developed by Liang et al. [28]. An efficient binary sand cat swarm optimization approach for feature selection in high-dimensional biomedical data was proposed by Pashaei [29], utilizing pinhole-imaging-based learning for cancer prediction. Finally, a recent review by Bulić et al. [30] highlighted how AI is increasingly shaping precision oncology by supporting multimodal diagnostics and personalized treatment optimization across multiple cancer types.

Cervical cancer diagnosis using an integrated system combining principal component analysis, genetic algorithm, and multilayer perceptron was reported by Dweekat and Lam [31]. Classification of cancerous and non-cancerous MRI using a dual DCNN approach was explored by Saeed et al. [32], comparing multiple architectures including InceptionV3, DenseNet121, and various ResNet. A summary of representative AI applications in oncology is provided in Table 1.

### 3.3. AI in Cardiovascular and Metabolic Disease Prediction

Several studies explored the use of deep learning and signal processing for cardiac and metabolic risk prediction. Yang et al. [33] analyzed isolated P-wave characteristics from 12-lead ECGs using machine learning to identify potential atrial fibrillation during sinus rhythm. Similarly, Khan Mamun et al. [34] demonstrated one-dimensional CNNs for cardiovascular risk classification based on clinical parameters, providing a practical method for wearable and EHR data integration.

Decoodt et al. [35] proposed hybrid classical–quantum (CQ) transfer learning models based on DenseNet-121 to detect cardiomegaly from chest X-ray images. Their models achieved strong performance, with an ROC-AUC of up to 0.93 and accuracy of up to 0.87, comparable to classical deep learning approaches. Decoodt et al. [36] presented transfer learning video classification of preserved, mid-range, and reduced left ventricular ejection fraction in echocardiography, utilizing AutoML approaches for cardiac function assessment. Lei et al. [37] developed hybrid decision support systems to monitor atrial fibrillation for stroke prevention, emphasizing human–AI collaboration and symbiotic analysis processes. Chen et al. [38] modeled hypertension and pregnancy outcomes, where maternal–neonatal prognostic models applied machine learning for preeclampsia risk estimation. Perišić et al. [39] investigated polygenic risk scores and risk factors for preeclampsia and gestational hypertension, integrating GWAS data with machine learning approaches. Prabhakar et al. [40] reported the use of machine learning for early detection of knee osteoarthritis and quantifying treatment effectiveness using force platform data, combining balance metrics with biomechanical analysis. Sohail et al. [41] presented an accurate clinical implication assessment for diabetes mellitus prevalence based on a study from Nigeria, employing data mining, cluster analysis, and various machine learning techniques including PART and decision tables using the Weka platform. Ahsan et al. [42] systematically evaluated the effect of data scaling methods on machine learning algorithms and model performance for heart disease prediction, providing methodological guidance for preprocessing strategies. Mariani et al. [43] explored analyzing medical data using statistical learning models, applying deep feedforward neural networks to heart disease, prostate cancer, and breast cancer datasets. A summary of selected AI models in this area is provided in Table 2.

### 3.4. AI for Infectious Disease Detection and Public Health

COVID-19 catalyzed a surge in AI-driven diagnostics. Latif et al. [44] used CNN models to distinguish novel coronavirus pneumonia from common pneumonia in chest CTs, emphasizing model generalization and transfer learning. Pradhan et al. [45] proposed a demographic-based AI model to predict COVID-19 positivity using SMOTE balancing and explainability techniques such as SHAP and LIME, ensuring interpretability in public health contexts. Le et al. [46] enhanced portable chest X-ray quality through deep learning, enabling fast COVID-19 monitoring in low-resource settings. Khaloufi et al. [47] developed deep learning-based early detection frameworks for preliminary diagnosis of COVID-19 via onboard smartphone sensors, enabling prediction using mobile device capabilities. Abbaspour et al. [48] investigated identifying modifiable predictors of COVID-19 vaccine side effects using machine learning, incorporating time-of-day effects and allergy history with model explanation techniques.

Cho and Hong [49] reported applying machine learning to healthcare operations management through CNN-based models for malaria diagnosis, addressing epidemic diagnosis. Their results demonstrate that machine learning can improve healthcare operations by enhancing diagnostic quality, speed, cost efficiency, productivity, and financial outcomes compared to manual methods. Khafaga et al. [50] presented an Al-Biruni Earth radius optimization-based deep convolutional neural network for classifying monkeypox disease, utilizing meta-heuristic optimization for skin disease detection, illustrating how nature-inspired optimization strategies can be effectively coupled with deep learning architectures for emerging infectious disease classification. A summary of representative AI applications in infectious disease management is provided in Table 3.

### 3.5. AI in Neurological and Cognitive Disorders

AI is increasingly leveraged to analyze complex neuroimaging and cognitive health data. Bangyal et al. [51] proposed a deep convolutional neural network-based approach for Alzheimer’s disease diagnosis, integrating ontology construction with deep learning knowledge. Mandal and Mahto [52] introduced a deep multi-branch CNN for early Alzheimer’s detection from brain MRIs, achieving enhanced sensitivity through multi-path feature fusion.

Huynh et al. [53] combined generative adversarial networks (GANs) with graph convolutional networks (GCNs) for neuroimaging classification based on resting-state functional MRI, highlighting the role of synthetic augmentation for small datasets. In addition, Zhang et al. [54] developed preclinical diagnosis of magnetic resonance brain images via discrete wavelet packet transform with Tsallis entropy and a generalized eigenvalue proximal support vector machine, demonstrating advanced feature extraction using entropy-based methods and kernel techniques for pattern recognition.

Ozkan [55] conducted a comparison of classification methods for telediagnosis of Parkinson’s disease, evaluating feature transformation techniques including principal component analysis and k-nearest neighbor approaches for telemedicine applications. Complementing this work, Dhillon et al. [56] developed a Raspberry Pi-based traumatic brain injury detection system for single-channel electroencephalogram, enabling low-cost, portable TBI screening. Lenkala et al. [57] presented a comparison of automated machine learning (AutoML) tools for epileptic seizure detection using electroencephalograms, evaluating performance across different AutoML platforms for time series analysis. In a related area of precision neurology, Guan et al. [58] investigated neuroimaging markers for studying Gulf War illness using single-subject level analytical methods based on machine learning, employing Kansas case criteria with diffusion imaging and neurite density assessment. In a related area of precision neurology, Pérez-Cano et al. [59] reported characterization of a clinically and biologically defined subgroup of patients with autism spectrum disorder and identification of tailored combination treatment, utilizing precision medicine approaches with metabolic and transcriptomic alterations analysis. A summary of representative AI applications in neurological and cognitive health is provided in Table 4.

### 3.6. AI in Medical Imaging and Computer Vision

AI-based medical imaging includes disease-specific diagnostics as well as general image processing and segmentation methods.

Zhang et al. [60] proposed an attentive octave convolutional capsule network for medical image classification, incorporating attention mechanisms with octave convolution for improved feature representation. Zou et al. [61] developed an interactive image segmentation method based on multi-level semantic fusion, balancing model complexity with cross-stage feature aggregation. Oghalai et al. [62] presented automated segmentation of optical coherence tomography images of the human tympanic membrane using deep learning, utilizing TensorFlow and CNNs for otologic applications. Abuhussein and Robinson [63] reported obscurant segmentation in long-wave infrared images using GLCM textures, employing unsupervised texture analysis with Gabor filters and Markov random fields. Extending these imaging techniques to respiratory disease, Jamjoom et al. [64] developed a Gaussian mixture with max expectation guide for stacked architecture of denoising autoencoder and DRBM for medical chest scans and disease identification, specifically targeting pneumonia prediction through Boltzmann machine architectures.

Collazo et al. [65] proposed a deep learning-based preprocessing and normalization approach for high-resolution whole-slide images, achieving substantial improvements in automatic region-of-interest labeling. They reported 845% dataset expansion and 96% reduction in expert annotation time, underscoring the transformative potential of automated preprocessing pipelines in reducing the bottleneck of manual expert annotation in large-scale pathology studies. A summary of representative AI applications in medical imaging and computer vision is provided in Table 5.

### 3.7. Algorithmic Innovations and Framework Development

Beyond clinical applications, several works contributed to algorithmic and theoretical frameworks that underpin healthcare AI adoption.

Rosenberg et al. [66] proposed an interpretable AI model using expressive Boolean formulas for improved transparency in clinical decision support, incorporating stochastic local search and large neighborhood search with potential quantum computing applications. Building on the theme of responsible AI, Soltan and Washington [13] examined fairness in breast cancer stage classification, introducing post-processing equalized-odds correction to mitigate algorithmic bias through equalized opportunity methods. Furthermore, Ghimire and Amsaad [67] presented a parallel approach to enhance the performance of supervised machine learning realized in a multicore environment, demonstrating ensemble model acceleration through multicore processing for improved accuracy and computational efficiency. Table 6 summarizes selected studies contributing to algorithmic transparency, fairness, and computational efficiency in healthcare AI.

### 3.8. Summary of Methodological Approach

Across the reviewed literature, most studies utilized supervised learning techniques, particularly convolutional neural networks and support vector machines, often enhanced through transfer learning or hybrid feature selection. Data sources spanned structured EHR records, imaging modalities (CT, MRI, ultrasound), and sensor data. Evaluation protocols predominantly included accuracy, sensitivity, specificity, and area-under-curve (AUC) metrics, although interpretability and generalizability were less consistently addressed. This synthesized review provides a methodological foundation for understanding how fragmented data architectures constrain AI scalability and informs the comparative policy and interoperability analyses presented in subsequent sections.

Table 7 summarizes the key findings across all reviewed clinical domains, highlighting the principal insights and recurring limitations identified in the literature.

The publications listed in Table 8 provide some key insights on concepts associated with data engineering as it applies to healthcare delivery. However, it must be noted that a key contribution of this narrative review is that here, the authors focus on conceptual challenges associated with data engineering as it applies to healthcare delivery, as listed in the objectives described in the Section 1.

## 4. Discussion

The literature reviewed in this study shows that the adoption of AI in U.S. healthcare is accelerating across many clinical domains, but progress is uneven because the supporting data infrastructure often lags behind model development. Across oncology, cardiovascular and metabolic risk prediction, infectious disease detection, neurological conditions, and medical imaging, researchers report strong performance for supervised learning approaches (especially deep learning), frequently using structured EHR variables, imaging modalities (CT, MRI, ultrasound), and sensor data [4,33,44,52,65]. However, these successes are often demonstrated in controlled settings with narrowly defined datasets, which does not always translate to reliable real-world deployment [11,13].

### 4.1. Data Engineering as the Practical Bottleneck Solution for Clinical AI

A consistent theme across application areas is that model performance depends less on the choice of algorithm than on the ability to build dependable data pipelines. Healthcare data encompasses large volumes of information drawn from multiple and diverse resource domains (notes, labs, images, devices), and they are created for clinical operations rather than analytics. As a result, data may be incomplete, inconsistently coded, difficult to access, or fragmented across departments and institutions [19]. These constraints affect dataset construction, labeling, longitudinal linkage, and the ability to reproduce results across sites. For example, Read et al. [19] demonstrated that longitudinal EHR data can support gastrointestinal cancer prediction, but only after extensive temporal feature engineering, highlighting that data preparation, not modeling, was the primary challenge. Similarly, Collazo et al. [65] showed that automated preprocessing pipelines can reduce expert annotation time by 96% and expand dataset size by 845%, underscoring how data engineering investments directly unlock AI scalability. Researchers commonly address these challenges through chart reviews or prospective data collection, approaches that require considerable time and resources. In practice, the most important barrier to scaling AI is often the absence of standardized processes for ingestion, cleaning, integration, and ongoing maintenance of datasets and metadata. From a technical standpoint, a typical healthcare AI data pipeline involves several critical stages: data extraction from heterogeneous sources (EHR systems, imaging repositories, laboratory databases, and wearable sensors); transformation steps including normalization, deduplication, missing value imputation, and format standardization; and loading into structured repositories suitable for model training, collectively referred to as Extract, Transform, Load (ETL) processes. Beyond ETL, data versioning—maintaining traceable, timestamped snapshots of datasets as they evolve—is essential for reproducibility and audit trails in clinical AI, yet it is rarely discussed explicitly in the reviewed literature. Data cleaning in healthcare is particularly challenging due to inconsistent coding practices, free-text clinical notes, and device-specific sensor formats [19]. Collazo et al. [65] demonstrated that investment in automated preprocessing and normalization pipelines can dramatically reduce the manual effort required to prepare data for model training, underscoring that the technical sophistication of data engineering steps, not just model architecture, determines whether AI systems can be reliably deployed and maintained over time. This can help with, for example, whether or not we must develop an AI-based system for cancer diagnosis. The quality and reliability of training data will be very critical. Also, this training data must be derived from various reliable sources to balance various elements of demography.

Nasir and Gurupur [72] have explained the importance of calculating completeness of electronic health records. In this seminal work, the authors promote the idea of quantifying completeness of electronic health records. This work was further elaborated upon by Nasir et al. [73], where data from the 2014 Florida Healthcare Cost and Utilization Project was used to identify areas of incompleteness in terms of education and other demographic variables. Furthermore, Gurupur and Shelleh [74] explained the use of machine learning and ontologies to predict incompleteness of electronic health records.

### 4.2. Risk, Liability, and Privacy Constraints Shape Technical Choices

Compared with many other industries, healthcare carries a higher consequence of failure. The potential for serious harm increases legal exposure and drives conservative deployment, especially when responsibility for errors is unclear. Studies such as Mobiny et al. [7], which employed Bayesian deep networks and Monte Carlo dropout to quantify model uncertainty in skin lesion diagnosis, illustrate how risk-awareness must be embedded directly into model design rather than treated as an afterthought. While AI models leverage multiple outcome measures to reduce the impact of data collection errors, this strategy alone may be insufficient to address fundamental data quality challenges [3].

Furthermore, privacy laws and the risk of litigation constrain data sharing and multi-institutional learning. These realities directly influence data engineering decisions: what can be stored, how it can be accessed, what must be de-identified, and whether models can be trained centrally or require privacy-preserving alternatives, such as federated learning. As a result, the “best” technical solution is often the one that fits regulatory and governance constraints while still supporting reliable data quality checks and auditing.

Here we would like to emphasize the need for regulatory standards in testing these AI systems for reliability. For example, if a cancer diagnosis system is developed using AI, there is a need to ascertain the acceptable level of accuracy. If the system fails to meet these standards, they must be barred from use. Here we must also identify the fact that the training data was accrued using the existing statutory regulations.

### 4.3. Evaluation Gaps: Interpretability, Generalizability, and Bias

Most studies rely on conventional performance metrics (accuracy, sensitivity, specificity, AUC) [42,43]. While these are necessary, they are not sufficient for high-stakes clinical use. Two gaps appear repeatedly. First, interpretability is addressed inconsistently, even though clinical decision support requires explanations that clinicians can understand and contest. Work on interpretable frameworks and explainability (e.g., expressive logic-based formulations) is promising [75], but it remains underrepresented relative to black-box performance reporting. Second, generalizability is frequently assumed rather than demonstrated. Many models are trained on single-site datasets or limited cohorts [24,26], and external validation is not always emphasized. These limitations are closely tied to data engineering realities: without consistent data standards, shared definitions, and careful documentation, models are difficult to validate across institutions.

In recent years, significant efforts have been devoted to integrating data from multiple institutions into unified databases, such as the All of Us Research Program and the One-Florida+ Clinical Research Network. In addition, large healthcare institutions increasingly utilize data collected from their multiple locations for AI modeling. Although integrated datasets continue to face challenges due to variations in clinical practices and data standards, these initiatives demonstrate that healthcare professionals recognize existing data gaps and are actively investing substantial effort into data integration pipelines.

Bias and fairness concerns further complicate deployment. When training data reflects unequal access to care, incomplete documentation, or demographic imbalances, models can reproduce and amplify these disparities. Studies that explicitly address bias mitigation (e.g., post-processing fairness methods) highlight that discovery and correction of bias requires not only algorithmic techniques but also data governance, subgroup evaluation, and transparent reporting [13]. From a data engineering perspective, fairness hinges on the responsible collection of relevant demographic and social context variables, the preservation of data provenance, and the ability to audit outcomes across subgroups without compromising privacy. When structural factors (e.g., access barriers, historical disadvantage, geography, disability status) are absent or only crudely represented, downstream models may appear neutral while continuing to reproduce underlying inequities. Accordingly, choices about which demographic and socioeconomic variables to collect, and at what level of granularity, directly shape both the feasibility and the integrity of fairness assessments. These choices, in turn, impose clear responsibilities on data collection practices, including: (1) gathering sensitive attributes explicitly to support auditing and harm detection rather than operational decision-making; (2) interpreting variables within their social and historical contexts rather than treating them as innate or static traits; and (3) clearly documenting why attributes are collected and how they will (not) be used. Finally, auditing data collection practices through logging, versioning, and review by both internal and external stakeholders helps ensure that fairness considerations are embedded from the earliest stages of the data lifecycle.

Here we need to be careful with the integration of different AI models. One example, is a situation where we have two different systems performing cancer diagnosis and we are trying to integrate the training generated by both these systems while not taking into consideration the fact that these discrete systems used different models and the fact that integration of the results may not be cohesive in nature.

### 4.4. Interoperability and “Fragmented Architectures” as a Core Systems Problem

A key implication of this review is that fragmented data architectures constrain AI scalability. Even when models achieve high performance in one dataset, as demonstrated by Dunn et al. [20] for lung cancer subtype classification and Shehata et al. [23] for renal cancer assessment, scaling to real clinical environments requires interoperability across EHR systems, imaging repositories, laboratory systems, and device platforms. Interoperability is not solely a standards issue; it also involves consistent ontologies, aligned data definitions, stable identifiers, version-controlled pipelines, and monitoring to detect dataset drift. At the standards level, interoperability frameworks provide a common data exchange format that enables disparate EHR systems, imaging platforms, and laboratory systems to communicate consistently, reducing the data integration burden upstream of AI pipelines [76]. For instance, HL7 Fast Healthcare Interoperability Resources (FHIR) is a modern HL7 standard that uses RESTful APIs and web technologies (JSON, XML) to enable granular, real-time access to healthcare data through standardized resources such as Patient, Observation, and Medication [77]. Furthermore, SMART on FHIR (Substitutable Medical Applications, Reusable Technologies on FHIR) is a profile and security framework that sits on top of the FHIR standard and enables third-party applications to run securely inside or alongside EHR systems using standardized APIs and authorization protocols, which streamlines data exchange.

For standardized communication, terminology and semantic standards in healthcare provide controlled vocabularies, code systems, and mappings that ensure that clinical data preserves its meaning when exchanged, stored, analyzed, or reused across systems and organizations [78]. Standards such as SNOMED CT for diagnoses and clinical concepts, LOINC for laboratory tests and clinical observations, RxNorm for medications, and ICD-10 for administrative reporting enable consistent and computable representations of health information. Together, they allow receiving systems to accurately interpret clinical intent rather than merely processing data structure, thereby supporting reliable interoperability, analytics, and clinical decision-making.

Finally, organizational interoperability is critical for enabling large-scale, multi-institutional research by allowing data to be shared securely and efficiently across organizations [79]. One prominent example is the Observational Medical Outcomes Partnership (OMOP), which defines a common data model (OMOP CDM) developed and maintained by the global OHDSI community to support large-scale observational health research and analytics. OMOP provides a standardized data structure, a unified set of clinical domains, and required terminology mappings that transform heterogeneous healthcare data—such as electronic health records, claims, and registries—into a consistent, analysis-ready format. By normalizing source data into common tables (e.g., person, condition, drug exposure, and measurement) and standard vocabularies, primarily SNOMED CT, RxNorm, LOINC, and mapped ICD codes, OMOP enables reproducible, multi-site studies, cohort definitions, and comparative effectiveness analyses without requiring institutions to share raw, patient-level data. OMOP is optimized for research, population-level analytics, and evidence generation, allowing the same analytical methods to be executed consistently across institutions and countries. Many times, we find out that systems used by different healthcare providers cannot communicate between one another. In some cases, different regions of a particular country use different EHRs that cannot communicate between each other, while, in other cases, the clinics and hospitals both belonging to the same organization use different EHRs that cannot communicate between them.

Together, improvements in these areas can reduce duplication of effort, improve reproducibility, and shorten the path from research prototypes to dependable clinical tools [65].

### 4.5. Future Directions

The authors intend to explore the following concepts as they relate to data engineering in healthcare in their future work:

- The idea of end-to-end data pipeline design with an emphasis on reusable data channels that support cleaning, labeling, provenance tracking, and continuous updates, rather than one-off dataset construction. Here we will investigate different approaches and methods used to successfully implement interoperability across healthcare ecosystems.
- The methods and techniques used for external validation by investigating the concept of multi-site evaluations and reporting performance across different healthcare ecosystems. This is especially true if data is gathered from different systems and the accrued data is used for decision-making in healthcare.
- The methods and techniques used for routine subgroup auditing, documentation of missingness, and bias checks into data engineering workflows. The concept of missingness and bias is rarely investigated in data engineering applied to healthcare.
- Approaches that enable learning from distributed data while respecting privacy constraints, paired with clear governance structures. Here an investigation on improving data governance while maintaining privacy and security is needed.

**Figure 3:**
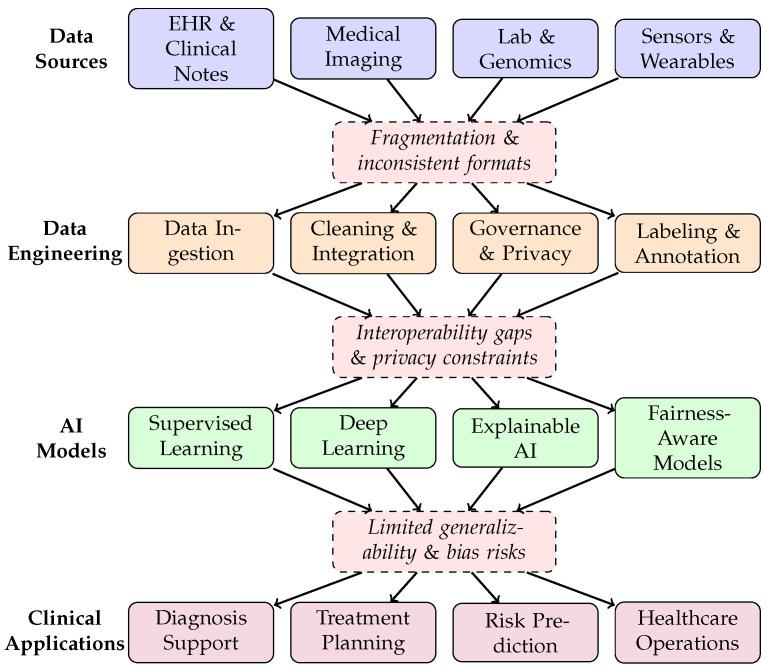
Conceptual diagram of the healthcare AI data pipeline, illustrating the flow from heterogeneous data sources through data engineering steps to AI models and clinical applications. Dashed boxes indicate key bottlenecks identified in the reviewed literature. Currently as the information flows through the pipeline they experience roadblocks (Fragmentation & inconsistent formats; Interoperability gaps & privacy constraints; Limited generalizability & bias risks) ultimately effecting the data quality and the quality of models reliant on this information.

Figure 3 provides a conceptual overview of the healthcare AI data pipeline, illustrating the flow from heterogeneous data sources through data engineering steps to AI models and clinical applications, with key bottlenecks annotated at each stage.

### 4.6. Limitations

This article was written based on the articles extracted as per the PRISMA diagram. While AI in healthcare is a large domain in this article, the authors limited the extracted articles, and the discussion based on the objectives delineated in the Section 1 mostly focused on data engineering.

## 5. Conclusions

Overall, the reviewed literature demonstrates that healthcare AI is advancing rapidly in scope and performance, but the ability to deploy these systems safely and at scale depends on data engineering maturity. Strengthening interoperability, governance, documentation, and evaluation practices will be essential to translate promising research results into trustworthy tools that improve patient outcomes. Although AI is accelerating across a wide range of clinical domains, its meaningful integration into real-world U.S. healthcare requires robust and reliable data engineering infrastructure. Persistent implementation challenges, such as fragmented data architectures, inconsistent documentation, privacy constraints, and limited interoperability continue to impede progress, creating a substantial gap between AI research and its practical deployment. Nonetheless, recent advances in data engineering, such as emerging multi-institutional data integration initiatives, highlight meaningful progress toward addressing existing barriers. Ultimately, strengthening end-to-end data pipelines, governance frameworks, and interoperability standards will be essential for enabling safe, equitable, and scalable deployment of AI tools capable of improving patient outcomes in the U.S. healthcare system. The key takeaways of this article in terms of adding to the body of discussion and perspective would be:

1. Identifying the need for reliable AI systems and problems associated with reliability,
2. Exploration of challenges within the healthcare ecosystem in adopting AI systems for healthcare, and
3. Identifying some of the key limitations of data engineering with respect to use of AI in healthcare.

## Figures and Tables

**Table 1:** Representative AI applications in oncology.

Article	Journal	Focus Keywords
Skin cancer detection with hybrid CNN features [6]	*Mathematics*	Deep learning; dermoscopy; CAD; CNN
Breast MRI radiomics for tumor classification [10]	*Diagnostics*	Radiomics; MRI; dynamic contrast; breast cancer
Renal cancer CAD system [23]	*Sensors*	CE-CT; morphology; texture; functionality
Multi-tumor biomarker tests with ML algorithms [27]	*Cancers*	Screening; health check-ups; machine learning

**Table 2:** Selected AI models for cardiovascular and metabolic disorders.

Article	Journal	Keywords
Atrial fibrillation prediction via ECG features [33]	*J. Pers. Med.*	Cardiovascular diagnosis; signal processing; EMD; ML
Preeclampsia and gestational hypertension risk [39]	*J. Pers. Med.*	Pregnancy; polygenic score; ML; GWAS
Knee osteoarthritis detection via force platform [40]	*JSAN*	Machine learning; balance metrics; biomechanics

**Table 3:** Examples of AI applications in infectious disease management.

Article	Journal	Key Concepts
COVID-19 detection from CT scans [44]	*Viruses*	Deep learning; image classification; CNN
Smartphone sensor framework for COVID prediction [47]	*Sensors*	On-device AI; mobile sensors; real-time inference
ML model for vaccine side-effect prediction [48]	*Vaccines*	Time-of-day effects; allergy; explainable ML

**Table 4:** Representative AI applications in neurological and cognitive health.

Article	Journal	Focus Keywords
Deep learning for Alzheimer’s MRI [52]	*Sensors*	Brain imaging; CNN; disease detection
GAN–GCN neuroimaging classification [53]	*Brain Sciences*	Resting-state fMRI; GAN; GCN; deep learning
Autism spectrum disorder subgroup characterization [59]	*Biomedicines*	Precision medicine; ASD; transcriptomics

**Table 5:** Representative AI applications in medical imaging and computer vision.

Article	Journal	Focus Keywords
Capsule network for medical image classification [60]	*Applied Sciences*	Attention mechanism; octave convolution; CNN
Interactive segmentation via semantic fusion [61]	*Sensors*	Image segmentation; multi-level features; deep learning
Whole-slide image preprocessing and normalization [65]	*Bioengineering*	WSI; annotation; pathology; preprocessing

**Table 6:** Selected studies on algorithmic frameworks and model explainability.

Article	Journal	Keywords
Explainable AI using Boolean logic [66]	*MAKE*	Interpretable ML; Boolean search; ILP; QUBO
Bias reduction in breast cancer classification [13]	*Algorithms*	Fairness; post-processing; equalized odds
Parallel learning performance in multicore systems [67]	*MAKE*	Ensemble model; multicore computing; performance

**Table 7:** Summary of key findings across clinical domains reviewed.

Clinical Domain	Methods	Key Finding	Limitation
Oncology	CNN, radiomics, transfer learning, ensemble classifiers	Deep learning and radiomics achieve strong diagnostic accuracy across cancer types; multimodal data integration further improves performance	Models trained on single-site or narrow imaging datasets; limited generalizability
Cardiovascular & Metabolic Disease	1D CNN, ECG signal processing, transfer learning, federated approaches	AI reliably detects arrhythmias and metabolic risk from ECG and clinical parameters; quantum–classical hybrid models show emerging promise	Most studies use structured EHR or single-modality signals; real-world deployment remains limited
Infectious Disease	CNN, SMOTE balancing, explainability tools (SHAP, LIME), mobile sensors	COVID-19 accelerated AI diagnostics in low-resource settings; explainability techniques are increasingly integrated to support public health decision-making	Heavy reliance on pandemic-era datasets; unclear generalizability to endemic or novel pathogens
Neurological & Cognitive Disorders	Deep CNN, GAN, GCN, wavelet transforms, AutoML	AI shows strong potential for early detection of Alzheimer’s, Parkinson’s, and epilepsy; synthetic data augmentation partially compensates for small neuroimaging datasets	Small and demographically homogeneous cohorts; limited external validation
Medical Imaging & Computer Vision	Capsule networks, attention mechanisms, semantic segmentation, denoising autoencoders	Automated preprocessing and annotation pipelines can dramatically reduce manual expert effort; segmentation methods generalize across organ systems	Computational cost and variability in imaging protocols across institutions
Algorithmic Frameworks	Explainable AI (Boolean logic, SHAP), fairness post-processing, parallel ensemble learning	Interpretability and bias correction are technically feasible but remain underutilized in clinical AI pipelines; fairness requires data governance, not just algorithmic fixes	Adoption of explainability and fairness tools is inconsistent across the field

**Table 8:** Summary of related publications on big data in healthcare.

Publication	Description
Dharavath [68]	The author focuses on the amount of data and systems associated with electronic health record systems.
Dash et al. [69]	The authors focus on different applications associated with big data within healthcare systems.
Palanisamy et al. [70]	The authors list and describe different data frameworks associated with healthcare systems across the globe.
Zhang et al. [71]	In this review article, the authors provide a description of different types of data that can be incorporated in developing machine learning models for healthcare decision-making.

## Data Availability

There is no research database to report. The data were obtained from publicly available sources.

## Abbreviations

The following abbreviations are used in this manuscript:

AI	Artificial Intelligence
AUC	Area Under Curve
CAD	Computer-Aided Diagnosis
CE-CT	Contrast-Enhanced Computed Tomography
CNN	Convolutional Neural Network
CQ	Classical-Quantum
CT	Computed Tomography
DCGAN	Deep Convolutional Generative Adversarial Network
DCNN	Deep Convolutional Neural Network
EHRs	Electronic Health Records
EMD	Empirical Mode Decomposition
GAN	Generative Adversarial Network
GCN	Graph Convolutional Network
GLCM	Gray-Level Co-occurrence Matrix
GWAS	Genome-Wide Association Studies
LIME	Local Interpretable Model-agnostic Explanations
ML	Machine Learning
MRI	Magnetic Resonance Imaging
ROC	Receiver Operating Characteristic
SHAP	SHapley Additive exPlanations
TBI	Traumatic Brain Injury
